# Polymorphism in NEDD4L Is Associated with Increased Salt Sensitivity, Reduced Levels of P-renin and Increased Levels of Nt-proANP

**DOI:** 10.1371/journal.pone.0000432

**Published:** 2007-05-09

**Authors:** Jonas Dahlberg, Lars-Olof Nilsson, Fredrik von Wowern, Olle Melander

**Affiliations:** Department of Clinical Sciences, Malmö University Hospital and Lund University, Malmö, Sweden; Institute of Human Virology, United States of America

## Abstract

**Objective:**

Neuronal precursor cell expressed developmentally down-regulated 4-like (NEDD4L) is a regulator of the amiloride-sensitive epithelial sodium channel (ENaC), thus a candidate gene for salt sensitivity. Carriers of an intact NEDD4L C2-domain, encoded by the NEDD4L rs4149601 (G/A) GG genotype, together with the C-allele of the NEDD4L rs2288774 (C/T) polymorphism have previously been shown to have increased blood pressure. Our aim was to test if genetic variation in NEDD4L is associated with increased salt sensitivity.

**Methods:**

39 normotensive subjects were studied. The difference in 24-hour systolic blood pressure after four weeks on 150 mmol/day NaCl intake and four weeks on 50 mmol/day NaCl was defined as salt sensitivity. The rs4149601 and rs2288774 polymorphisms were genotyped using PCR-based techniques.

**Results:**

Carriers of the rs4149601 GG-genotype together with the rs2288774 CC-genotype had significantly higher salt sensitivity (median, IQR) (18.0, 7.5–20.0 mmHg vs 6.0, 0.0–10.0 mmHg, P = 0.007) and lower plasma renin concentration (P-renin) (6.0, 2.0–9.5 mU/L vs 15.0, 9.0–24.0 mU/L, P = 0.005) as compared to non-carriers of these genotypes. In carriers of the rs4149601 GG-genotype together with the rs2288774 CC- or CT-genotype, as compared to non-carriers, salt sensitivity was (8.0, 6.0–18.0 mmHg vs 5.0, 0.0–10.0 mmHg, P = 0.07) and P-renin (9.0, 6.0–16.0 mU/L vs 15.0, 9.0–28.0 mU/L, P = 0.03).

**Conclusion:**

Genetic NEDD4L variation seems to affect salt sensitivity and P-renin in normotensive subjects, suggesting that genotyping of NEDD4L may be clinically useful in order to identify subjects who benefit from dietary salt restriction in the prevention of hypertension.

## Introduction

Hypertension is a major risk factor for cardiovascular morbidity and mortality and affects 27% of the adult Swedish population [1]. Blood pressure has a normal distribution in the population suggesting a multifactorial background of blood pressure regulation and hypertension development. Genetic factors might explain 30–40% of population blood pressure variation [2] and the genetic component is most likely composed of many individual genetic variants. Environmental risk factors such as stressful lifestyle, lack of physical exercise, high caloric intake and salt-rich diet also contribute to elevated blood pressure and development of hypertension. Furthermore, the environmental risk factors are likely to interact with the genetic susceptibility variants. Given the complex etiology and pathophysiology of hypertension, the search for genetic variants of importance for blood pressure has been suggested to be more comprehensible by dissecting an “intermediate phenotype” of blood pressure such as salt sensitivity. Such a phenotype is plausibly influenced by substantially fewer genes and environmental factors [3]. Salt sensitivity, i.e. the individual blood pressure reaction to a given change in salt intake is a continuous trait [4] and cut-off limits used to dichotomize the trait are arbitrary and have differed between studies. The heritability of systolic salt sensitivity calculated in Black pedigrees has been shown to be as high as 74% suggesting a strong genetic component of this trait [5]. In addition, hypertensive patients are more salt sensitive than normotensive subjects [4] and normotensive subjects with heredity for hypertension have increased salt sensitivity [6], indicating that salt sensitivity is partially related to an inherited predisposition for hypertension.

Salt restriction at the population level, with the aim to reduce population blood pressure, is difficult to achieve [7], [8] and its ability to lower the incidence of cardiovascular disease is controversial. However, identifying and intervening with salt restriction in the most salt sensitive segment of the population would probably be a very cost-effective way of treating and preventing hypertension in this particular subset of the population. In a clinical context it is cumbersome to measure salt sensitivity. It therefore seems essential to identify bio-markers for salt sensitivity and thus provide physicians a manageable tool of whom to give intense educational dietary advice. We have previously shown that salt sensitivity, defined as the difference between blood pressure after one week of high (240 mmol daily) and one week of low (10 mmol daily) salt intake is directly correlated to N-terminal atrial naturetic peptide (Nt-proANP) and inversely correlated to plasma renin activity [9], [10]. In a study recently completed, “Salt Reduction to Avoid Hypertension” (SARAH), we found that salt sensitivity defined as the difference between blood pressure after four weeks of high (150 mmol daily) and four weeks of low (50 mmol daily) salt intake, i.e. a more clinically relevant intervention, was directly correlated to Nt-proANP and inversely correlated to plasma concentration of renin (P-renin) [11]. These studies thus suggest that renin and Nt-proANP may be used to predict salt sensitivity.

It has been proposed that salt sensitivity is related to the inability of the kidney to excrete sodium [12], [13]. Most monogenic forms of human hypertension are caused by mutations increasing renal sodium reabsorption primarily at the level of the amiloride sensitive sodium channel (ENaC) [14], [15]. These rare forms of hypertension are further characterized by extreme salt sensitivity and suppressed P-renin. Although salt sensitivity and the accompanying P-renin suppression we have observed at the population level is usually far from as extreme as that seen in monogenic forms of hypertension, the clinical resemblance between them suggests that salt sensitivity may have partially similar pathophysiology as monogenic forms of hypertension. In one of the monogenic forms of hypertension, Liddle's syndrome, activating ENaC mutations affect the PY motif of ENaC with the consequence that ENaC becomes insensitive to down regulation by a protein coded by the Neural precursor cell Expressed Developmentally Down-regulated 4 Like (NEDD4L) gene [16], [17]. Normally the NEDD4L protein interacts with the PY motif and regulates the cell surface expression of ENaC by ubiquination, thereby affecting the rate of sodium reabsorption in the distal nephron.

We recently showed that a combination of two common single nucleotide polymorphisms (SNP:s) (rs4149601 and rs2288774) located in the NEDD4L gene is associated with blood pressure variation in a population study from Malmö, Sweden [18]. The G→A substitution of the rs4149601 polymorphism at the first nucleotide of exon 1 of the NEDD4L gene leads to an alternative splice site, which generates a transcript encoding a protein lacking the functionally crucial Ca^2+^-dependent lipid binding domain (C2 domain) [19]. NEDD4L and its paralog NEDD4 lacking the C2 domain, down-regulate ENaC more potently than NEDD4L with an intact C2 domain [16], [20]–[22] suggesting that carriers of the NEDD4L rs4149601 G-allele have higher ENaC expression and higher renal sodium reabsorption through ENaC than carriers of the A-allele. In consequence, we found that subjects with the GG genotype of rs4149601, as compared with carriers of the AA-genotype, had higher diastolic blood pressure [18]. Furthermore, subjects with the GG-genotype simultaneously carrying at least one copy of the C-allele of the NEDD4L rs2288774 T→C polymorphism in intron 6 had significantly higher systolic and diastolic blood pressure compared to non-carriers of this genotype combination, suggesting that carriers have increased tubular sodium reabsorption due to the decreased capacity of NEDD4L to down regulate ENaC. As expected in a highly complex and polygenic trait as blood pressure, the effect of the genotype combination on systolic and diastolic blood pressure was moderate, approximately 2/1 mmHg [18].

Given our previous findings and the well-described interaction between NEDD4L and ENaC, our aim was to elucidate whether the NEDD4L rs4149601 GG-genotype in combination with at least one C-allele of the NEDD4L rs2288774 polymorphism would be associated with increased salt sensitivity, reduced levels of P-renin and increased levels of Nt-proANP.

## Methods

The protocol of the study was approved by the ethics committee of Lunds University, and all study participants gave written informed consent. The procedures were in accordance with institutional guidelines.

### Subjects

Forty-six subjects with no history of hypertension, diabetes or kidney diseases were recruited through newspaper advertisements. Of these, 7 did not complete the study because of infections with fever (n = 2) or refusal to take the study capsules regularly (n = 5). Thus, 39 healthy subjects (53±11 years of age, BMI 26.3±3.1 kg/m^2^, number of men/women 20/19) completed the study. Other phenotype characteristics are presented in Table 1.

**Table 1:** 

	Baseline	High salt	Low salt	P* (high-vs. low-salt)
24-hour SBP (mmHg)	139±13.3	136±12.7	131±11.1	<0.0001
24-hour DBP (mmHg)	86.3±7.4	85.0±7.0	82.3±6.6	0.004
24-hour HR (bpm)	72.3±9.0	67.7±8.4	69.1±7.9	0.08
Daytime SBP (mmHg)	144±13.2	141±12.8	135±11.7	<0.0001
Daytime DBP (mmHg)	90.6±7.8	89.2±7.3	86.9±6.9	0.02
Daytime HR (bpm)	76.1±9.7	67.7±8.4	73.8±9.1	0.04
Night-time SBP (mmHg)	129±14.4	128±13.6	121±11.5	<0.0001
Night-time DBP (mmHg)	77.5±7.9	76.4±7.6	73.0±7.9	0.002
Night-time HR (bpm)	64.4±9.0	58.4±8.2	59.5±6.9	0.17
Body weight (kg)	79.5±11.2	77.4±10.7	77.3±10.6	0.43
Urine-Na^+^ (mmol/24h)	165±67.4	140±39.5	50.7±17.3	<0.0001
Urine-K^+^ (mmol/24h)	75.0±22.9	50.8±11.3	50.9±14.1	0.94
Urine-Crea (mmol/24h)	13.1±3.3	12.3±2.7	12.2±3.0	0.65
Serum-Na^+^ (mmol/L)	140±1.8	141±1.5	139±1.7	<0.0001
Serum-K^+^ (mmol/L)	3.7±0.26	3.6±0.25	3.7±0.30	0.21
Serum-Crea (mmol/L)	78.4±12.8	80.4±12.8	84.1±12.6	0.001
Plasma renin (mU/L)	15.3±9.4	17.4±11.2	32.4±18.4	<0.0001

Refer to individual change of variable on high- as compared with on low-salt (i.e. variable Δ-value). SBP, systolic blood pressure; DBP, diastolic blood pressure; Crea, Creatinine; HR, heart rate.

### Design

All the subjects were first examined at baseline, i.e. with the subjects on their regular diets before the standardization of salt intake started. During a period of eight weeks the subjects were given all their meals and drinks from a metabolic ward. Energy intake was adjusted according to body weight and gender (2000–2600 kcal/day). Apart from the provided meals and drinks, subjects were prohibited to ingest anything else apart from tap water. The diet during the eight weeks of study contained 50 mmol of salt (NaCl) daily. On top of this, the study participants were given either 100 mmol of salt in capsules (totally 150 mmol of salt daily) for four weeks and corresponding number of placebo capsules (totally 50 mmol of salt daily) for four weeks in a random order double blind crossover design [11].

Ambulatory blood pressure (ABP) and 24-hour urinary excretion of sodium were measured at baseline, after the four weeks on the high salt intake (150 mmol daily) and after the four weeks of low salt intake (50 mmol daily). ABP was measured using an ABPM 90207 device (Spacelabs Medical Inc, Redmond, WA, USA), which was applied on their left arm using appropriate cuff sizes according to arm circumference. During the daytime period (6 am–10 pm) blood pressure was recorded every 20 min and during the nighttime period (10 pm–06 am) every 60 min. Subjects were advised to relax the arm and keep it along the body during blood pressure measurements. Urine was collected during the same 24-hour as the ABP measurements.

Salt sensitivity was defined as the difference between 24-hour systolic ABP after the four weeks on high salt intake and 24-hour systolic ABP after the 4 weeks on low salt intake. In addition, the salt induced change in diastolic ABP (diastolic salt sensitivity) was recorded and systolic and diastolic salt sensitivity were further divided into the daytime and nighttime periods. P-renin and Nt-proANP were measured at baseline in the upright position without prior rest using RIA diagnostic kits as described previously [9], [10]. Urine and serum concentrations of sodium, potassium and creatinine were measured by standard biochemical methods at baseline, after the high- and low salt intake periods.

### Genotyping

DNA was extracted from venous whole blood by standard methods [23]. Two variants in the NEDD4L gene (rs4149601, G→A in exon 1 and rs2288774, T→C, in intron 6) were genotyped using PCR. The polymorphisms were analysed using forward primer 5′-GCTTTCCTTTAATGCACTAAACCTTTAATATTGT-3′ and reverse primer 5′-GGTAAGACTTTGCTTGGTGGGG-3′ (rs4149601) and forward primer 5′-ACAGTCTCATGTTTGATGCTTCGT-3′ and reverse primer 5′-AGAAGGCTGAAATGAAGCACGT-3′ (rs2288774) synthesized by Applied Biosystems (Applied Biosystems, Foster City, California, USA). TaqMan MGB probes were custom synthesized by Applied Biosystems: (rs4149601, G) FAM-ATTTGAGCAGGTAACAC, (rs4149601, A): VIC-CATTTGAGCAAGTAACAC and (rs2288774, T) FAM-CACTCTGAAAATACACTGACT, (rs2288774, C) VIC-CACTCTGAAAATACGCTGACT, according to standard recommendations for the ABI Prism 7900HT analysis system (Applied Biosystems, Foster City, California, USA). Genotypes were determined by end-point fluorescent measurements [24].

### Statistics

All data in the study was analyzed with SPSS statistical software (version 11.5, SPSS Inc. Chicago, Illinois, USA). Data is presented as mean±standard deviation (SD) for normally distributed variables and as medians and interquartile ranges (IQR) for variables that were not normally distributed. Significance of group-wise and pair-wise differences in continuous variables was tested with t-test/paired t-test or Mann-Whitney/Wilcoxońs paired rank test depending on whether data was normally distributed or not. P<0.05 was considered statistically significant.

## Results

The rs4149601 (exon 1) and rs2288774 (intron 6) polymorphisms were tested separately and in combination for association to salt sensitivity indices and to baseline levels of Nt-proANP and P-renin.

Carriers of the rs4149601 GG-genotype had significantly lower P-renin than carriers of the rs4149601 GA or AA genotypes whereas no other phenotype differed significantly by genotype (Table 2). There was no association between any of the phenotypes and the rs2288774 polymorphism (Table 2).

**Table 2:** Salt-induced changes in blood pressure and baseline P-renin and Nt-proANP in different genotype carriers.

	rs4149601 genotype variants			
	GG (n = 16)	GA (n = 20)	AA (n = 3)	P-value; GG vs GA or AA
**Δ24SBP**(mmHg)	6.5(3.0–13)	6.5(0.0–10)	2.0(−8.0–15)	0.315
**Δ24DBP**(mmHg)	1.5(−1.0–9.75)	3.5(−0.5–5.75)	−1.0(−5.0–8.0)	0.703
**ΔDSBP**(mmHg)	6.5(2.0–14.5)	6.0(−2.0–9.0)	5.0(−6.0–7.0)	0.343
**ΔDDBP**(mmHg)	2.0(−3.0–9.75)	2.0(−1.0–4.75)	1.0(−5.0–3.0)	0.921
**ΔNSBP**(mmHg)	11.5(2.25–15)	6.0(0.25–9.0)	−3.0(−15–30)	0.107
**ΔNDBP**(mmHg)	4.5(1.25–9.5)	4.0(−2.75–7.0)	−4.0(−8.0–16.0)	0.196
**P-renin**(mU/L)	10.5(6.5–15.8)	18.0(10.0–28.0)	9.0(2.0–25.0)	0.045
**Nt-proANP**(pmol/L)	510(419–803)	510(422–646)	620(440–786)	0.641
	**rs2288774** genotype variants			
	**CC** (n = 12)	**CT** (n = 14)	**TT** (n = 13)	**P-value; CC vs CT or TT**
**Δ24SBP**(mmHg)	7.5(3.0–16.5)	4.0(−1.25–7.75)	7.0(2.5–12)	0.168
**Δ24DBP**(mmHg)	4.5(−0.5–8.25)	1.0(−2.0–4.0)	3.0(−1.0–6.5)	0.221
**ΔDSBP**(mmHg)	7.5(2.75–19)	3.5(−2.0–7.0)	7.0(0.5–11.0)	0.178
**ΔDDBP**(mmHg)	3.0(0.25–9.75)	−0.5(−1.5–4.0)	3.0(−2.0–5.5)	0.188
**ΔNSBP**(mmHg)	9.0(0.25–15)	4.5(−1.0–8.75)	9.0(3.5–12.5)	0.443
**ΔNDBP**(mmHg)	5.5(−2.75–8.0)	3.5(−2.25–7.0)	5.0(2.0–7.5)	0.845
**P-renin**(mU/L)	10.5(6.5–19.5)	14.0(9.0–21.5)	14.5(6.75–28.75)	0.185
**Nt-proANP**(pmol/L)	659(438–844)	559(359–732)	489(436–599)	0.150

Blood pressure change in low versus (vs) high salt intake; Δ24SBP, delta 24-hour systolic blood pressure; Δ24DBP, delta 24-hour diastolic blood pressure; ΔDSBP, delta daytime systolic blood pressure; ΔDDBP, delta daytime diastolic blood pressure; ΔNSBP, delta night-time systolic blood pressure; ΔNDBP; delta night-time diastolic blood pressure. P-value<0.05 is considered statistically significant. Numbers represent Median(Inter Quartile Range).

Carriers of the rs4149601 GG-genotype together with the rs2288774 CC-or CT-genotype, as compared to non-carriers of this combination, displayed significantly lower P-renin and borderline significance for systolic salt sensitivity (P = 0.06–0.08) whereas there was no significance in diastolic salt sensitivity and Nt-proANP (Table 3).

**Table 3:** Salt-induced changes in blood pressure, and baseline P-renin and Nt-proANP in different genotype carriers.

	rs4149601 GG+rs2288774 CC/CT **genotype variants vs non-carriers**		
	GG+CC/CT (n = 11)	Non-carriers (n = 28)	P-value
**Δ24SBP**(mmHg)	8.0(6.0–18)	5.0(0.0–10)	0.067
**Δ24DBP**(mmHg)	2.0(−1.0–11)	2.0(−1.0–5.75)	0.158
**ΔDSBP**(mmHg)	8.0(4.0–19)	5.0(−2.0–8.75)	0.058
**ΔDDBP**(mmHg)	4.0(−3.0–11)	1.5(−1.0–3.75)	0.140
**ΔNSBP**(mmHg)	14.0(2.0–15)	5.5(0.0–9.0)	0.083
**ΔNDBP**(mmHg)	8.0(1.0–10)	4.0(−2.75–7.0)	0.124
**P-renin**(mU/L)	9.0(6.0–16)	15.0(9.0–28.0)	0.030
**Nt-proANP**(pmol/L)	754(426–880)	493(422–622)	0.109
	**rs4149601 GG+rs2288774 CC** genotype vs non-carriers		
	**GG+CC** (n = 5)	**Non-carriers** (n = 34)	**P-value**
**Δ24SBP**(mmHg)	18(7.5–20)	6.0(0.0–10)	0.007
**Δ24DBP**(mmHg)	9.0(0.5–13.5)	1.5(−1.25–6.0)	0.081
**ΔDSBP**(mmHg)	19(5.0–21.5)	5.0(−2.0–9.0)	0.024
**ΔDDBP**(mmHg)	9.0(0.0–13.5)	1.5(−1.0–4.0)	0.117
**ΔNSBP**(mmHg)	15.0(12.5–20)	5.0(−0.25–9.5)	0.001
**ΔNDBP**(mmHg)	8.0(4.5–11)	4.0(−2.0–7.0)	0.089
**P-renin**(mU/L)	6.0(2.0–9.5)	15.0(9.0–24.0)	0.005
**Nt-proANP**(pmol/L)	806(590–1186)	493(417–655)	0.031

Blood pressure change in low versus (vs) high salt intake; Δ24SBP, delta 24-hour systolic blood pressure; Δ24DBP, delta 24-hour diastolic blood pressure; ΔDSBP, delta daytime systolic blood pressure; ΔDDBP, delta daytime diastolic blood pressure; ΔNSBP, delta night-time systolic blood pressure; ΔNDBP; delta night-time diastolic blood pressure. P-value<0.05 is considered statistically significant. Numbers represent Median(Inter Quartile Range).

**Figure 1:**
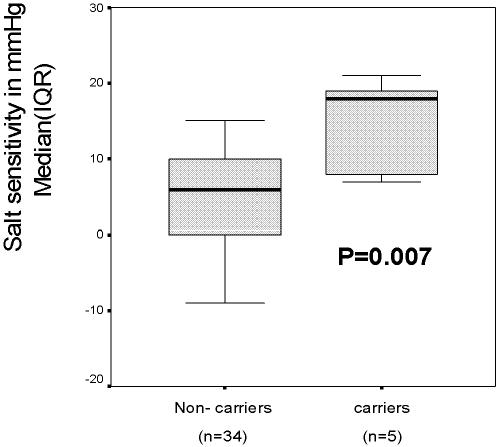
Salt induced change in 24 hour systolic ABP (salt sensitivity) in the carriers of the rs4149601 GG-genotype together with the rs2288774 CC-genotype vs. all other genotype combinations. Box plot presented as median and inter quartile range.

Carriers of the rs4149601 GG-genotype together with the rs2288774 CC-genotype had significantly higher 24-hour, daytime and nighttime systolic salt sensitivity, lower P-renin and higher Nt-proANP when compared with non-carriers (Fig 1 and Table 3), whereas the differences in indices of diastolic salt sensitivity were not statistically significant.

## Discussion

The key finding of this study is that the carriers of the rs4149601 GG-genotype together with rs2288774 CC-genotype have enhanced systolic salt sensitivity, lower P-renin and higher Nt-proANP compared with subjects who do not carry this genotype combination of the NEDD4L gene.

NEDD4L and its paralog NEDD4 lacking the functionally crucial C2-domain down-regulate ENaC more potently than the protein variants with intact C2-domain [16], [20]–[22]. As the rs4149601 A-allele alters a splice site leading to preferential deletion of the C2 domain [19], subjects carrying the G-allele would be expected to have a less efficient NEDD4L induced down regulation of ENaC and thus increased renal sodium reabsorption. Interestingly, carriers of the rs4149601 GG-genotype had significantly lower P-renin but did not differ regarding salt sensitivity (Table 2), indicating reduced activity of the renin-angiotensin-aldosterone system (RAAS) as a response to a slight increase in renal sodium reabsorption in order to counterbalance the effect of the GG-genotype on salt sensitivity. Carrier ship of the rs2288774 CC-genotype seemed to amplify the effect of the rs4149601, thereby unmasking clinically detectable salt sensitivity. In contrast to the rs4149601, the functional consequence of the rs2288774 at the molecular level is unknown. The NEDD4L gene is highly polymorphic suggesting that the rs2288774 polymorphism in intron 6 could be non-functional but be in linkage disequilibrium with another functional NEDD4L variant. Interestingly, intronic SNP:s in the transcription factor 7-like 2 (TCF7L2) gene have been strongly associated with another complex phenotype, that of type 2 diabetes mellitus, in numerous populations of various ethnicities [25]. Thus, another possibility is that rs2288774 is functional, despite being intronic. Such functional mechanisms may involve interference with intronic transcription factor binding sites, leading to altered NEDD4L expression, or introduction of a cryptic splice site. Importantly, in a recent study on genetic NEDD4L variance and blood pressure in subjects without antihypertensive treatment, we found that the rs4149601 polymorphism seems to interact with another NEDD4L polymorphism, rs2288774 [18]. In that study carriers of the rs4149601 GG-genotype together with rs2288774 CT or CC-genotypes had approximately 2/1 mmHg higher office blood pressure compared to non-carriers [18]. Carriers of the same genotype combination in the present study tended to have higher systolic salt sensitivity and had significantly lower P-renin (Table 3). On the other hand, carriers of the rs4149601 GG-genotype together with rs2288774 CC-genotype, who had the greatest systolic salt sensitivity in the present study (Table 3), did not have significantly higher blood pressure in the previous population study [18]. Although related, it is important to stress that blood pressure and salt sensitivity are different phenotypes. The relatively greater suppression of baseline P-renin in carriers of the rs4149601 GG-genotype together with rs2288774 CC-genotype compared to that seen in carriers of rs4149601 GG-genotype together with rs2288774 CT or CC-genotypes (Table 3) may level out the effect on blood pressure through RAAS-dependent mechanisms other than those directly affecting renal sodium reabsorption. Despite the substantially smaller material used in the present study, as compared with the previous population study on blood pressure, there were several reasons to believe that we would have power enough to detect an effect of the NEDD4L polymorphisms on salt sensitivity. Most importantly, salt sensitivity is a much less complex phenotype than blood pressure. Blood pressure is influenced by numerous environmental factors such as stress, physical activity, caloric intake and level of salt intake. Although salt sensitivity may also be affected by a number of environmental factors, the standardized phenotype of salt sensitivity used in the present study is primarily influenced by the shift of salt intake from 150 mmol to 50 mmol daily and the individual inherited and acquired capacity of handling changes in salt intake. As fewer environmental factors are implicated in salt sensitivity than in blood pressure, it can be speculated that fewer genes conferring gene-environment interactions are involved in salt sensitivity. In addition, whereas heritability of blood pressure is usually around 30–40%, that of systolic salt sensitivity has been reported to be as high as 74% [5], suggesting a substantially greater genetic component of systolic salt sensitivity than for blood pressure. Finally, in our previous study on blood pressure, the phenotype used was office blood pressure, whereas in the present study of salt sensitivity the more accurate blood pressure phenotype of ABP was used to define blood pressure after the two different levels of salt intake. Taken together, the effect of genetic variance of proteins involved in the process of renal sodium reabsorption, such as NEDD4L, is likely to be much greater on the phenotype of salt sensitivity than on population blood pressure.

P-renin and Nt-proANP measured under non-standardized conditions of salt intake have earlier been shown to predict salt sensitivity [9], [19]. A likely explanation for this is that salt sensitivity is a state of sodium hyper-reabsorption at the level of the renal tubules, leading to suppression of RAAS due to increased flow of sodium through the juxtaglomerular apparatus of the kidney and to increased atrial secretion of Nt-proANP as a consequence of atrial wall distension. This potential mechanism as the cause of enhanced salt sensitivity is supported by the pathophysiology of monogenic forms of human hypertension, such as Liddlés syndrome [14], in which over-expression of ENaC in the distal nephron leads to hyper-reabsorption of sodium, volume expansion, suppression of RAAS and extremely salt sensitive hypertension. Thus, the associations between the NEDD4L rs4149601 and rs2288774 polymorphisms on the one hand and P-renin and Nt-proANP on the other, are likely to be secondary to genetically mediated enhancement of tubular sodium reabsorption rather than being a direct effect of the genetic variants on renin and Nt-proANP secretion.

Our associations were generally stronger for systolic than for diastolic salt sensitivity (Tables 2 and 3). This is probably explained by the fact that the heritability of systolic salt sensitivity is substantially higher than for diastolic salt sensitivity [5] giving us a greater power to detect genetic factors influencing systolic salt sensitivity.

In summary, we show that carriers of the rs4149601 GG-genotype together with rs2288774 CC-genotype of the NEDD4L gene have enhanced systolic salt sensitivity, lower P-renin and higher Nt-proANP. Our data suggests that carriers of this genotype combination have increased renal sodium reabsorption through reduced NEDD4L induced down-regulation of ENaC. As salt sensitivity differs a lot between individuals, genotyping of these genetic NEDD4L markers may be a clinically useful tool in identifying those individuals who would gain the greatest blood pressure lowering benefit from reduced dietary salt intake.
